# Angiogenic Secretion Profile of Valvular Interstitial Cells Varies With Cellular Sex and Phenotype

**DOI:** 10.3389/fcvm.2021.736303

**Published:** 2021-08-30

**Authors:** Victoria Nelson, Vaidehi Patil, LaTonya R. Simon, Kelsey Schmidt, Chloe M. McCoy, Kristyn S. Masters

**Affiliations:** ^1^Department of Biomedical Engineering, University of Wisconsin-Madison, Madison, WI, United States; ^2^Department of Medicine, University of Wisconsin School of Medicine and Public Health, Madison, WI, United States; ^3^Department of Materials Science and Engineering, University of Wisconsin-Madison, Madison, WI, United States

**Keywords:** sexual dimorphism, valvular endothelial cells, calcific aortic valve disease, valvular interstitial cell, angiogenesis, thrombospondin

## Abstract

Angiogenesis is a hallmark of fibrocalcific aortic valve disease (CAVD). An imbalance of pro- and anti-angiogenic factors is thought to play a role in driving this disease process, and valvular interstitial cells (VICs) may act as a significant source of these factors. CAVD is also known to exhibit sexual dimorphism in its presentation, and previous work suggested that VICs may exhibit cellular-scale sex differences in the context of angiogenesis. The current study sought to investigate the production of angiogenesis-related factors by male and female VICs possessing quiescent (qVIC) or activated (aVIC) phenotypes. Production of several pro-angiogenic growth factors was elevated in porcine aVICs relative to qVICs, with sex differences found in both the total amounts secreted and their distribution across media vs. lysate. Porcine valvular endothelial cells (VECs) were also sex-separated in culture and found to behave similarly with respect to metabolic activity, viability, and tubulogenesis, but male VECs exhibited higher proliferation rates than female VECs. VECs responded to sex-matched media conditioned by VICs with increased tubulogenesis, but decreased proliferation, particularly upon treatment with aVIC-derived media. It is likely that this attenuation of proliferation resulted from a combination of decreased basic fibroblast growth factor and increased thrombospondin-2 (TSP2) secreted by aVICs. Overall, this study indicates that VICs regulate angiogenic VEC behavior via an array of paracrine molecules, whose secretion and sequestration are affected by both VIC phenotype and sex. Moreover, strong sex differences in TSP2 secretion by VICs may have implications for understanding sexual dimorphism in valve fibrosis, as TSP2 is also a powerful regulator of fibrosis.

## Introduction

Calcific aortic valve disease (CAVD) is the most common type of heart valve disease in the Western world (1). It is associated with mild to severe valve leaflet thickening and calcification and is driven by active pathobiological processes including chronic inflammation, fibrosis, and angiogenesis (1, 2). CAVD is more prevalent in men and exhibits sexual dimorphism in its presentation, where females with CAVD have more fibrosis, while males have more calcification (3–5).

Healthy aortic heart valves are not vascularized, so any angiogenesis in the valve is considered pathological. Angiogenesis is thought to be both a consequence of CAVD and a factor in its progression. During early stages of CAVD, valve thickening causes diffusion limitations that can signal the need for vascularization. These vessels can then act as a source of stimuli that further regulate pathological processes in the valve, such as chronic inflammation (6). Valvular endothelial cells (VECs) line the blood-contacting surfaces of the valve and may serve as a source of cells for valvular neovascularization (7). Imbalances in pro- and anti-angiogenic factors are thought to be a driving factor in their dysfunction (8) and, ultimately, the development of angiogenesis seen in CAVD (6).

Meanwhile, valvular interstitial cells (VICs) are the dominant cell type throughout the bulk of the valve and are believed to play a role in mediating these angiogenic processes. VICs are active in secreting soluble biomolecules and synthesizing extracellular matrix (ECM) components to regulate and remodel the valve environment (9, 10). VICs are primarily present in a quiescent (qVIC) phenotype in the healthy aortic valve, and they can be activated to a myofibroblastic, or activated (aVIC), phenotype in response to injury or pathological insult (10). Recent work has shown that VICs isolated from human valves with CAVD display pro-angiogenic properties and can differentiate into a perivascular phenotype (11).

Crosstalk between VICs and VECs has been described in the context of VIC homeostasis and disease, and VICs can stimulate angiogenic sprouting by VECs (7). However, relatively little remains known about paracrine angiogenic interactions between VICs and VECs and how they vary with VIC phenotype. Additionally, the crosstalk between these cells has not been studied in the context of cellular-scale sex differences. A previous microarray analysis of male and female VICs found that angiogenesis was one of seven biological process categories overrepresented in male VICs (12), suggesting that VIC behavior in the context of angiogenesis could differ with sex. Moreover, no previous studies have examined whether VEC behavior is dependent upon cellular-scale sex. These gaps in knowledge motivated the current work, wherein we quantify the secretion of angiogenic factors by both male and female qVICs and aVICs and then characterize the effects of these paracrine factors on angiogenesis-related activity of sex-matched VECs. Improved characterization of angiogenesis in CAVD could lead to a better understanding of CAVD pathogenesis and could inform the development of pharmacological CAVD treatments.

## Methods

### VIC Isolation

Valvular interstitial cells (VICs) were isolated from male and female porcine aortic valves (Hoesly's Meats, New Glarus, WI) and cultured separately by sex. Aortic valve leaflets were excised from porcine hearts, and VICs were immediately isolated via collagenase digestion, as described previously (13). The cells were then plated on a 10 cm tissue culture plate, refed with growth medium on Day 1 and then every other day after that until they reached 70-90% confluency. Cells were then passaged onto either normal tissue culture plates to continue as activated VICs (aVICs) or collagen-coated plates to culture into quiescent VICs (qVICs), according to published protocols (14). Briefly, qVICs were generated via culture in low-glucose DMEM supplemented with 2% FBS, 100 U/mL penicillin (P4333; Sigma), 100 μg/mL streptomycin (P4333; Sigma), bFGF (10 ng/mL; Peprotech, Rocky Hill, NJ) and 5.25 mg/mL insulin (I0516; Sigma). Generation of qVIC vs. aVIC cultures was validated by qRT-PCR for alpha smooth muscle actin (ACTA2), where aVICs were confirmed by a minimum 10-fold increase in ACTA2 gene expression as previously described (14). All VICs were refed every other day and subcultured until passage three, at which point they were seeded for experiments. For each sex, each experiment used VICs pooled from *N* = 3 pigs; all experiments were repeated a minimum of two times, with each repeat using N=3 pigs that did not overlap with previous donor pools. All VICs were used as fresh isolates and never frozen down.

### VEC Isolation

Valvular endothelial cells (VECs) were isolated from male and female porcine aortic valves (Hoesly's Meats, New Glarus, WI) and cultured separately by sex. Aortic leaflets were dissected and washed twice in an M199 solution containing 100 U/mL penicillin (P4333; Sigma), 100 μg/mL streptomycin (P4333; Sigma), and 200 mM L-glutamine solution (G7513; Sigma). The leaflets were then incubated in a 37°C water bath in a PBS solution including 60 U/mL collagenase type 2 (Worthington Biochemical Corp., Lakewood, NJ), penicillin (100 U/mL), and streptomycin (100 μg/L) for 120 minutes. Leaflets were separated from the enzyme solution by passing through a 100 μm cell strainer and then added to PBS pre-warmed to 37°C and vortexed for 60 seconds to dislodge the VECs from the tissue matrix. The cell solution was separated from the leaflets by passing through a 100 μm cell strainer and pelleted via centrifugation at 1,000 rpm for 10 min. The cell pellet was resuspended in 10 mL EGM-2 basal growth medium (CC3162; Lonza), and the cells were then plated on 10 cm plates coated with 2% gelatin in sterile deionized water for expansion. After expansion, cells were prepared for fluorescence-activated cell sorting (FACS). Briefly, cells were detached using TrypLE (ThermoFisher), pelleted, and then resuspended in FACS buffer (0.5% BSA, 2 mM EDTA, 20 mM HEPES in PBS). Cells were counted, incubated with FITC-conjugated anti-CD-31 (5 μL per 10^6^ cells, MCA1746F; BioRad, Hercules, CA) at 4°C for 30 mins, counterstained with DAPI (1 μg/mL), and washed. FACS was performed using a BD FACSAria (BD Biosciences, Franklin Lakes, NJ) to select for live cells positively stained for CD-31. VECs were used between passages three and seven. Each batch of VECs was from a single donor, and each experiment was repeated 2-4 times, using a new donor each time (for a total of *N* = 3–5 separate donors, with *n* = 3 samples/condition per donor).

### Angiogenic Growth Factor Secretion Profile of VICs

Male and female aVICs and qVICs were seeded in 10 cm dishes at a density of 50,000 cells/cm^2^ and cultured in standard growth medium (low-glucose DMEM with 10% FBS and 1% penicillin-streptomycin solution) for 48 h. The aVICs were cultured on 10 cm tissue culture coated plates while qVICs were cultured on 10 cm collagen-coated plates in order to retain their qVIC phenotype for the duration of the experiment. Seven ELISAs were run on the conditioned culture media and culture lysate (collected in RIPA buffer, 89900; ThermoFisher): endothelin-1 (DY1160; R&D Systems, Minneapolis, MN), platelet derived growth factor-A (PDGF-A, DY221; R&D), vascular endothelial growth factor-A (VEGF-A, DY293B; R&D), basic fibroblast growth factor (bFGF, DY233; R&D), epidermal growth factor (EGF, DY236; R&D), insulin-derived growth factor-1 (IGF-1, DY291; R&D), and thrombospondin-2 (TSP2, DTSB20; R&D), all per manufacturer's instructions. The media and lysate were differentially diluted in the sample dilution buffer specific to each ELISA kit such that all sample absorbance readings fell within the dynamic range of each ELISA. A Quant-iT™ PicoGreen™ dsDNA Assay (P11496; Thermo Fisher Scientific) was performed on the lysate to normalize to cell number across all samples.

### Heparan Sulfate *in situ* ELISA

Heparan sulfate proteoglycan-2 (HSPG2) deposition was assayed by semi-quantitative immunocytochemical detection. Cells were fixed in 10% neutral buffered formalin (Sigma), endogenous peroxidase activity was quenched with 0.3% hydrogen peroxide in methanol for 1 h, and samples blocked overnight in 3% goat serum. Samples were then incubated with monoclonal mouse anti-HSPG2 antibody (ab23418, Abcam, Waltham, MA) for 2 h followed by a series of washes with 1X PBS and labeling with a horseradish peroxidase-linked goat anti-mouse secondary antibody (polyclonal, 0.5 μg/mL; Sigma) for 40 min. After washing, samples were incubated in 1-Step Turbo-TMB ELISA substrate solution (ThermoFisher) and the reaction stopped after 5 min through the addition of 2 N sulfuric acid (ThermoFisher). Color development was proportional to HSPG2 content and measured with a plate reader. Background signal was determined by following the same procedure without incubating in primary antibody solution. This background absorbance was subtracted from the samples for the respective culture condition. Corrected absorbance values were normalized to total cell number determined by DAPI staining.

### VEC Proliferation

Proliferation of VECs at baseline and in response to VIC-conditioned media was analyzed using a Click-iT EdU cell proliferation assay (C10337; Thermo Fisher Scientific). Male and female aVICs and qVICs were seeded at 50,000 cells/cm^2^ in 10 cm plates and cultured as described above, with conditioned media collected after 48 h. VIC growth medium was also placed in dishes without cells for 48 h to generate a matched negative control comprised of unconditioned, but incubated, media. Male and female VECs were seeded at 21,000 cells/cm^2^ in 48-well plates coated with 2% gelatin. VECs were allowed to grow for 8 h prior to refeeding with VIC-conditioned (or unconditioned) media, and then cultured overnight. VECs were incubated with EdU solution for 2 h and then fixed with 4% paraformaldehyde. Detection of EdU was performed following manufacturer instructions with a Hoechst^®^ 33342 counterstain. Images were taken on a Zeiss Axiovert.A1 inverted microscope and analyzed manually, with percent proliferation expressed as the number of EdU-positive cells divided by the total cell number in each field of view at three fields of view per well and with three wells per condition. Proliferation experiments were also repeated in the presence of a neutralizing antibody to CD36, which acts as the receptor for TSP-2 (Clone FA6-152, Stem Cell Technologies, Vancouver, Canada), or an isotype control (mouse IgG1 K, 550878, BD Biosciences, Franklin Lakes, NJ), which were added to VECs at a concentration of 5 μg/mL for 30 min prior to refeeding with conditioned medium.

### VEC Tubulogenesis

Male and female aVICs and qVICs were seeded in 10 cm dishes at a density of 50,000 cells/cm^2^ and cultured in standard growth medium as described above. Conditioned media was collected from VICs after 48 h of culture. VIC growth medium was also placed in dishes without cells for 48 h to generate a matched negative control comprised of unconditioned, but incubated, media. Male and female VECs were seeded at 62,000 cells/cm^2^ in 96-well plates coated with 8–11 mg/mL growth factor-reduced Matrigel (354230; Corning) and allowed to attach for 30 min. The conditioned VIC media was then applied to VECs in a sex-matched manner (i.e., media from male VICs applied to male VECs). The VECs were cultured in the VIC-conditioned (or unconditioned) media for 4 h prior to analyzing tubulogenesis. The positive control was cultured in EGM-2 media supplemented with VEGF to ensure that the cells at that density would form tubes. Images were taken at three fields of view per well and with three wells per condition on the Zeiss AxioVert.A1 inverted microscope and analyzed through WIMASIS (Córdoba, Spain).

### VEC Viability and Metabolic Activity

To assess VEC viability, male and female VECs were seeded at 10,000 cells/well in a 96 well plate and cultured for 72 h. Cells were then washed in PBS and incubated for 30 min in Live/Dead stain (L3224, ThermoFisher) at concentrations of 2 μM Calcein AM and 4 μM EthD-1. Cells were then imaged on a Zeiss Observer Z1, with three images taken per well, *n* = 3 wells per condition. Images were analyzed using Fiji with a macro from Allevi by 3D Systems to count live and dead cells; viability is expressed as % live cells. VEC metabolic activity was measured using CellTiter-Glo (G7570, Promega, Madison, WI). For this assay, male and female VECs were seeded in opaque-walled 96 well plates at a density of 10,000 cells/well and cultured for ~72 h. Cells were equilibrated at room temperature for 30 min prior to adding 100 μL CellTiter-Glo solution to the 100 μL cell culture medium already present in the wells. The plates were placed on an orbital shaker for 2 min, incubated statically for 10 min at room temperature for the signal to stabilize, and then luminescence read on a plate reader.

### Western Blot

Male and female aVICs and qVICs were cultured as described above for 48 h, at which point cell lysates were collected using Pierce RIPA buffer. A microBCA assay (ThermoFisher) was conducted to determine protein concentration for each sample. Samples were diluted to the same protein concentration and then denatured in a 70°C water bath for 10 min with Novex™ Tris-Glycine SDS Sample Buffer (LC2676; ThermoFisher). The samples were loaded into a 4–12% Tris-glycine gel and electrophoresis performed using a XCell SureLock Mini-Cell system (EI0001; Thermo Fisher Scientific) was run at 200 V for 30 min. The gel was blotted onto a PVDF membrane overnight at 4°C at 16 V. The membrane was blocked in 5% w/v milk in PBST for 90 min at room temperature and then incubated with anti-chondromodulin-1 antibody (MAB41681, R&D) at a dilution of 1:1,000 at 4°C overnight. The membrane was then washed in TBST and incubated with an HRP conjugated secondary antibody at a dilution of 1:10,000 for 1h at room temperature, followed by washing with TBST, exposing to the ECL substrate, and imaging on the Bio-Rad ChemiDoc XRS imaging system (1708265; Bio-Rad).

## Results

### Angiogenic Factor Secretion Profile Varies With Cell Sex and Phenotype

**Figure 1:**
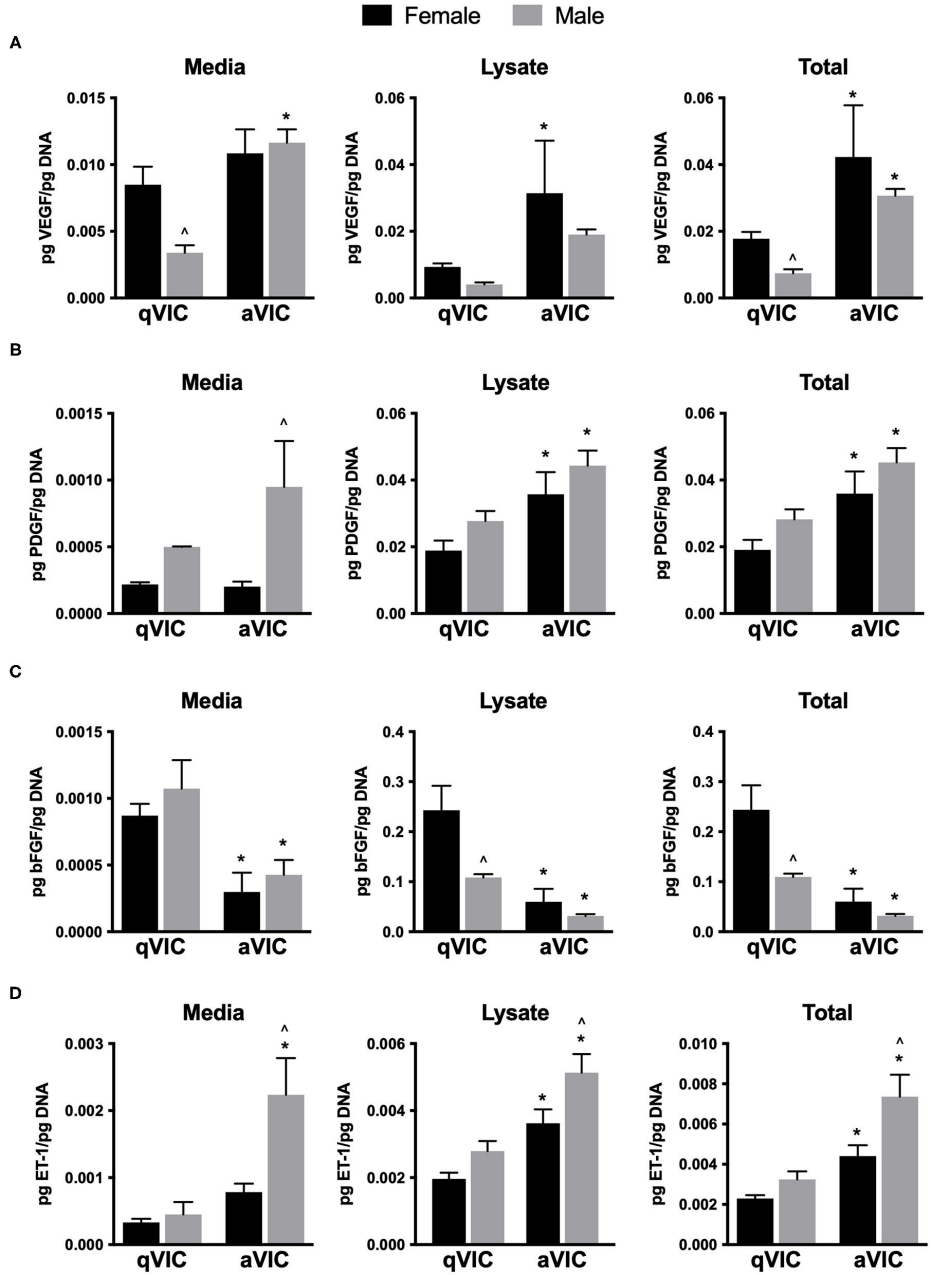
Production of pro-angiogenic factors by male and female qVICs and aVICs. **(A)** vascular endothelial growth factor (VEGF), **(B)** platelet derived growth factor-A (PDGF), **(C)** basic fibroblast growth factor (bFGF), and **(D)** endothelin-1 (ET-1). *p < 0.05 vs. sex-matched qVIC level; ^∧^*p* < 0.05 vs. female VIC of same phenotype by two-way ANOVA with Tukey's post *hoc* test. *N* = 3 pigs per sex, *n* = 3 wells per condition.

VIC secretion of seven factors known to participate in the promotion of angiogenesis was analyzed by ELISA. VEGF is the most commonly studied pro-angiogenic growth factor and was detected in the media and lysate of male and female qVICs and aVICs (Figure 1A). Sex differences in VEGF secretion were evident in qVICs only, with female qVICs secreting more VEGF than males. In general, the aVIC phenotype was associated with increased VEGF production by both male and female cells. A similar phenotype-dependent trend was observed for PDGF. For this growth factor, greater amounts of PDGF were found in aVIC cultures for each sex, compared to their qVIC counterpart (Figure 1B). However, PDGF secretion varied with sex for only one condition, where male aVICs had more PDGF in the media fraction compared to female aVICs.

bFGF (or FGF-2) is another growth factor that is a potent mitogen and pro-angiogenic agent (15). VIC secretion of bFGF tended to follow an opposite trend than what was observed for VEGF and PDGF, in that an activated VIC phenotype was associated with decreased bFGF production (Figure 1C). Although media levels of bFGF did not vary with sex, significantly less bFGF was found in the lysate of male qVICs compared to female qVICs, leading to a difference in bFGF totals between male and female qVICs. Endothelin-1 is not as commonly examined as a direct pro-angiogenic factor, but can be a potent EC mitogen (16); it was included in our study because an initial qualitative angiogenic factor screen (not shown) showed high levels of ET-1 in the media. Similar to the results described for VEGF and PDGF, ET-1 production tended to be significantly increased in aVICs compared to qVICs, and this was true across both sexes (Figure 1D). Additionally, a consistent sex-dependent effect was observed, where male aVICs produced more ET-1 than female aVICs.

Finally, EGF and IGF-1 round out the suite of factors that are frequently associated with pro-angiogenic actions (17, 18). These two molecules were not consistently found in detectable quantities in both the media and lysate of the cell phenotypes/sexes examined. Specifically, EGF was not present within the detection limits of the assay, while IGF-1 was found in female qVIC media and male qVIC lysate, although at the lower assay detection limit in both.

### Sequestration of Angiogenic Factors Varies With Cell Sex and Phenotype

**Figure 2:**
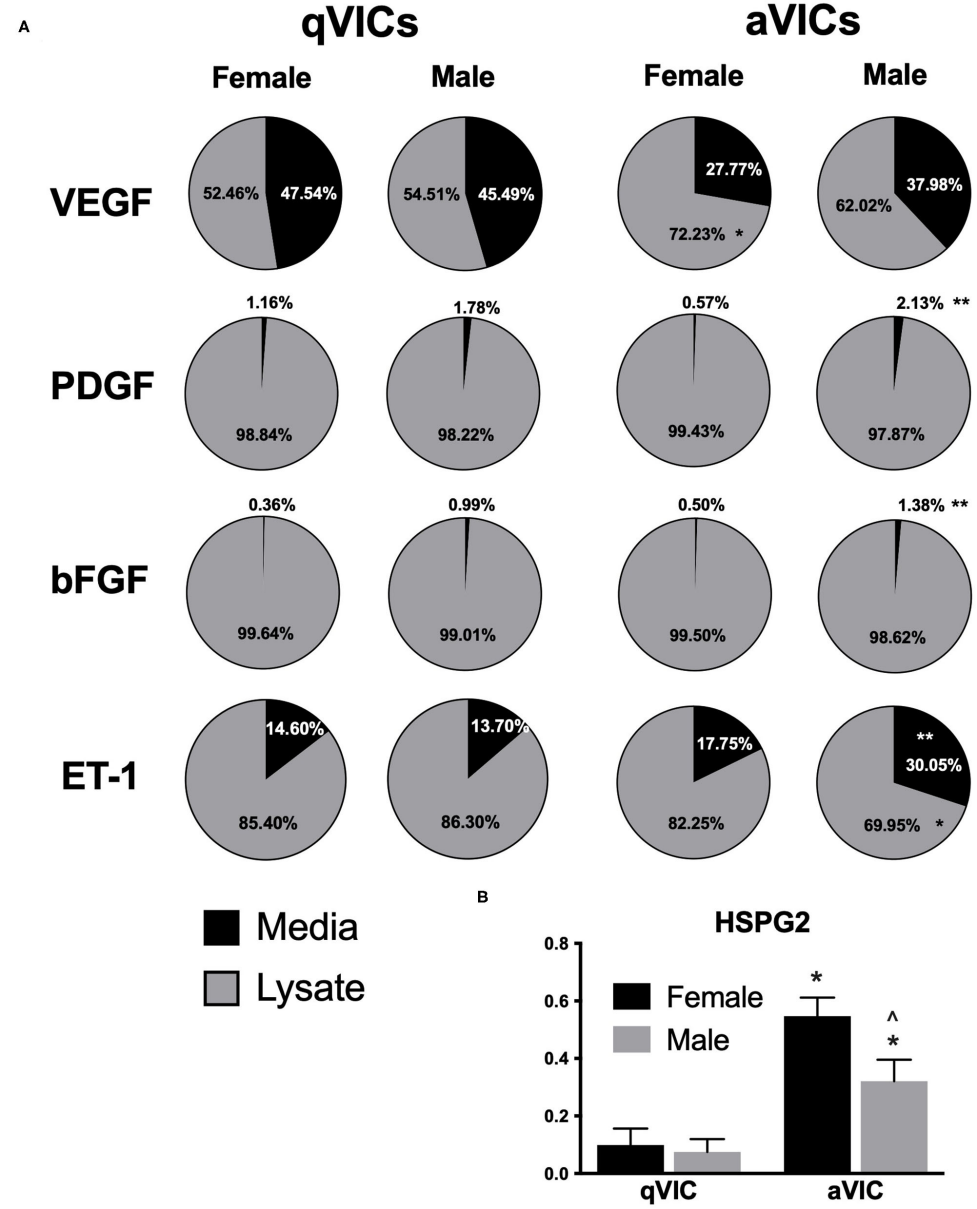
Distribution of pro-angiogenic factors across media and lysate from male and female qVICs and aVICs. **(A)** Percent of total growth factor found in media vs. lysate for VEGF, PDGF, bFGF, and ET-1. *p < 0.05 vs. sex-matched qVIC level; **p < 0.05 vs. female VIC of same phenotype. **(B)** Heparan sulfate proteoglycan 2 (HSPG2) found in extracellular matrix of male and female qVIC and aVIC cultures. *p < 0.05 vs. sex-matched qVIC level; ^∧^*p* < 0.05 vs. female VIC of same phenotype by two-way ANOVA with Tukey's post *hoc* test. *N* = 3 pigs per sex, *n* = 3 wells per condition.

Examination of the distribution of growth factors between media and lysate also uncovered sex- and phenotype-dependent trends (Figure 2A). Amongst qVICs, the fraction of each pro-angiogenic factor present in the media remained similar across both males and females. However, this was not the case for aVICs; for 3 of the 4 factors detected (PDGF, bFGF, ET-1), male aVICs had a significantly increased proportion of the molecule present in the media compared to female aVICs.

An alternative way of describing this change is that male aVICs had smaller amounts of sequestered growth factors relative to female aVICs. Because growth factor sequestration is highly dependent upon the extracellular matrix environment, we also evaluated whether sexual dimorphism in ECM secretion could be influencing these results. Heparan sulfate proteoglycans are generally responsible for growth factor binding (19), so an *in situ* ELISA for HSPG2 was performed. The HSPG2 ELISA results were consistent with the growth factor secretion profiles (Figure 2B); specifically, male aVIC cultures had less HSPG2 than female aVIC cultures, which could contribute to the lower amounts of sequestered factors in the male aVIC cultures.

### Baseline Valvular Endothelial Cell Behavior Exhibits Little Sex-Dependence

**Figure 3:**
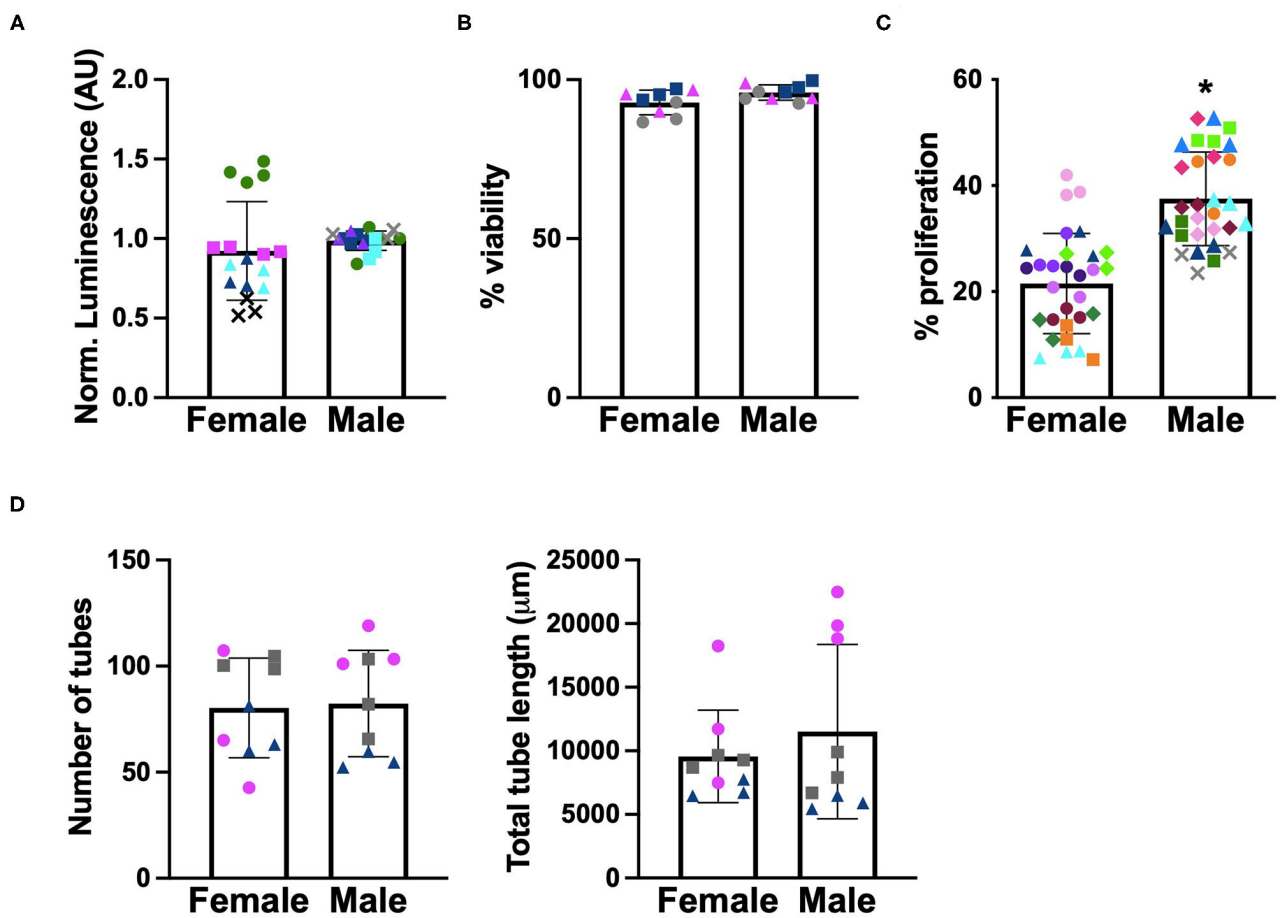
Sex differences in baseline characterization of VECs. **(A)** ATP activity, **(B)** viability, **(C)** proliferation, and **(D)** tubulogenesis. Different marker shapes indicate different donors; different colors within the same marker shape indicate different runs with the same donor. *p < 0.05 vs. female VECs by Welch's *t*-test. *N* = 3–4 pigs per sex, *n* = 3 wells per condition.

Before we could examine the impact of angiogenic factors secreted by male and female VICs on the function of sex-matched VECs, we first needed to characterize whether VECs exhibited sex-dependent angiogenic behaviors at baseline. Because VECs have not previously been separated by sex in culture, basic cell viability and metabolic activity were also quantified. As shown in Figures 3A,B, neither ATP activity nor viability were affected by cellular-scale VEC sex. Proliferation and *in vitro* tubulogenesis were then evaluated as measures more directly related to angiogenic activity. The proliferation of male VECs tended to be significantly higher than the proliferation of female VECs (Figure 3C), while tubulogenesis was similar across male and female VECs (Figure 3D).

### VIC-Conditioned Media Increases Tubulogenesis but Decreases VEC Proliferation

**Figure 4:**
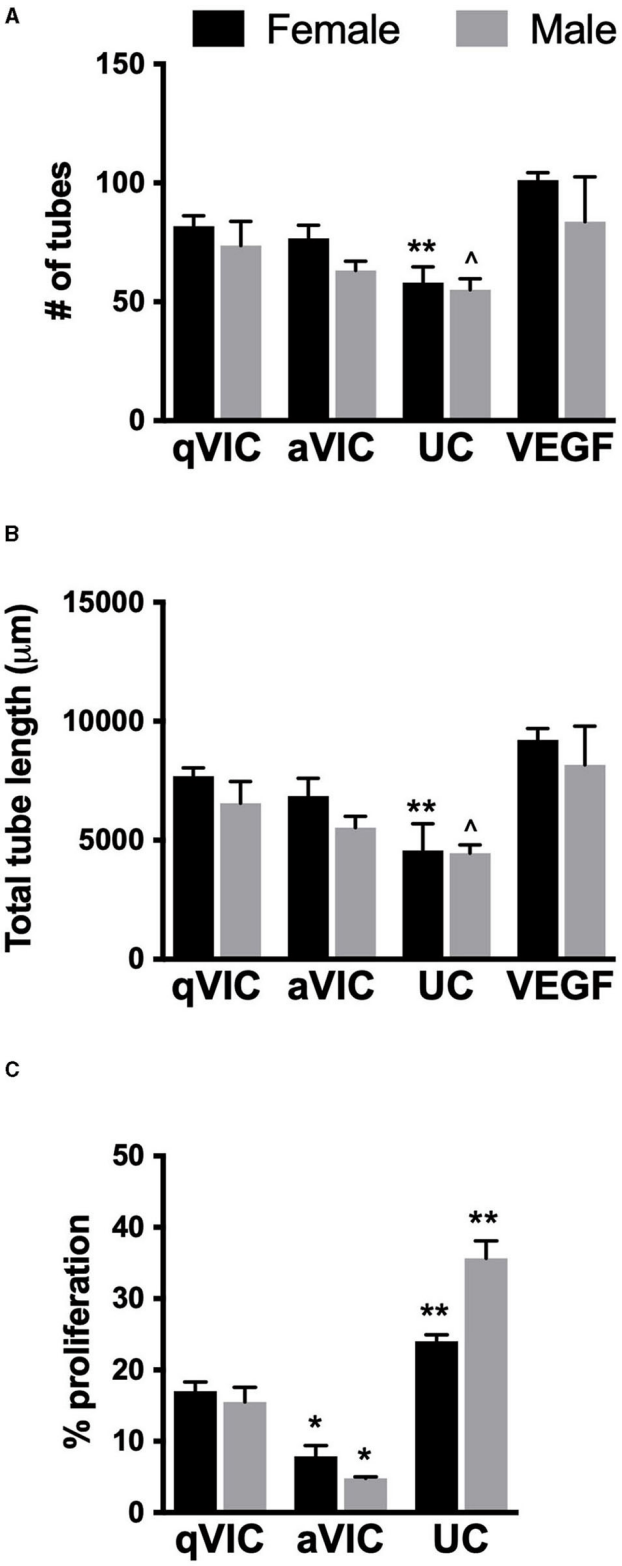
Effect of VIC-conditioned media on VEC function. **(A)** Number, **(B)** length of tubules formed by VECs in tubulogenesis assay, and **(C)** VEC proliferation. *p < 0.05 vs. sex-matched qVIC condition; **p < 0.05 vs. sex-matched aVIC and qVIC conditions by two-way ANOVA with Tukey's post *hoc* test. UC = unconditioned media. *N* = 3–4 pigs per sex, *n* = 3 wells per condition.

As demonstrated in Figure 1, VICs secrete numerous pro-angiogenic factors, so we hypothesized that VIC-conditioned media would promote angiogenic outcomes in VECs. This hypothesis appeared to hold true for tubulogenesis, where media conditioned by VICs tended to promote formation of more and longer VEC tubules compared to unconditioned media (Figures 4A,B). Sex differences were not observed in these results, suggesting that the differences in angiogenic secretion profiles of male and female VICs were not sufficient to drive sexual dimorphism in downstream events.

aVICs are the dominant VIC phenotype in diseased valves, and this phenotype tended to secrete more of these factors than qVICs in our study. Thus, we had also hypothesized that media conditioned by aVICs would show greater ability to increase angiogenic activity in VECs compared to media conditioned by qVICs. However, this was not the case, as tubulogenesis in VEC cultures treated with aVIC media was typically lower than when VECs were treated with qVIC media.

Analysis of VEC proliferation after treatment with VIC-conditioned media also yielded somewhat unexpected results. Specifically, all VIC-conditioned media decreased VEC proliferation relative to the unconditioned control (Figure 4C). Proliferation was lowest in VECs treated with aVIC-conditioned media. Given that the baseline (unconditioned media) proliferation of male VECs is significantly higher than female VECs, the magnitude of the decrease in proliferation upon application of aVIC-conditioned media was greater for males (8-fold) than for females (3-fold).

### Anti-angiogenic Factors Are Secreted by VICs

**Figure 5:**
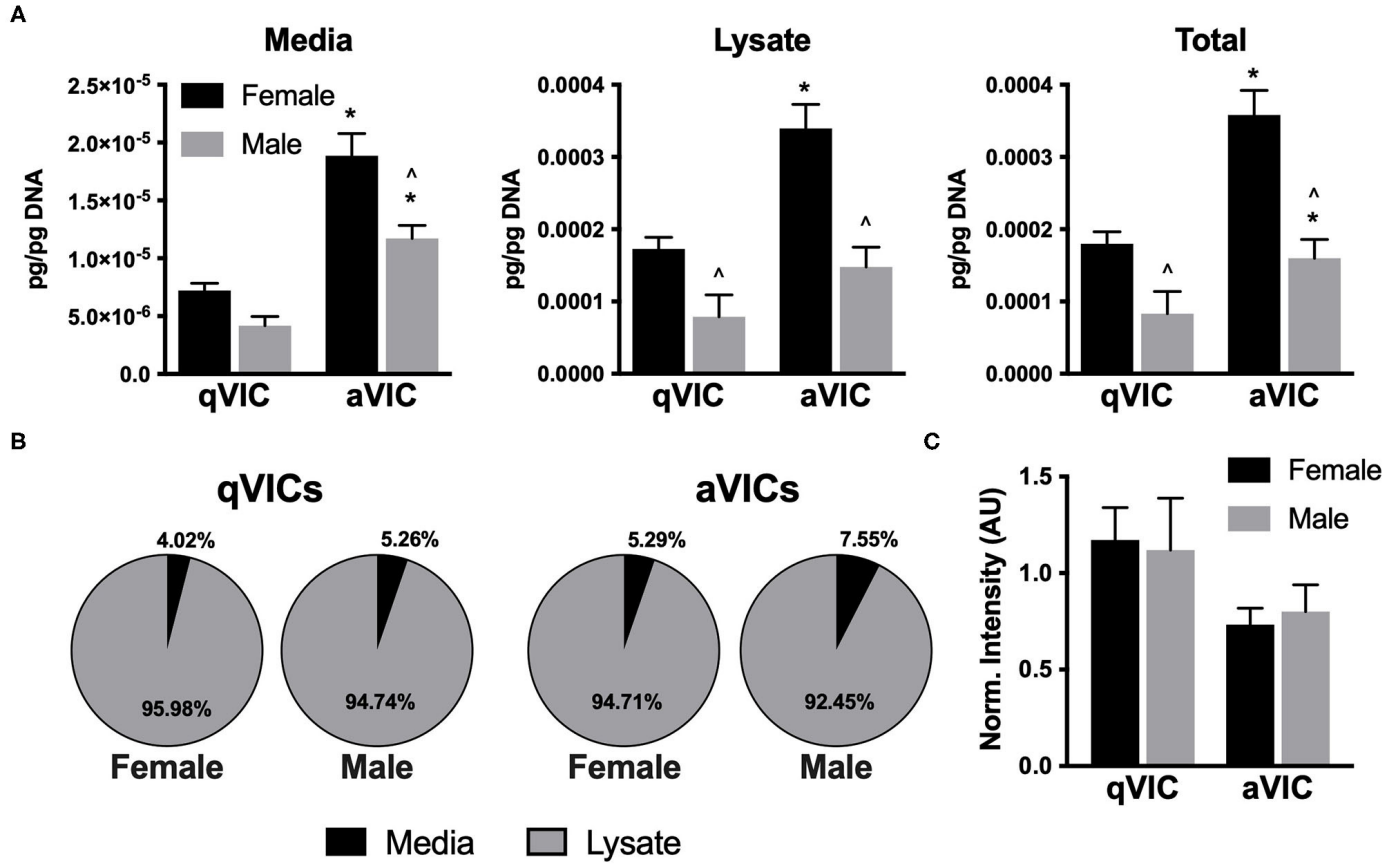
Production of anti-angiogenic factors by VICs. **(A)** Production and **(B)** distribution of thrombospondin-2 (TSP2) in VIC cultures. **(C)** Western blot quantification of chondromodulin-1 in VIC lysates. *p < 0.05 vs. sex-matched qVIC level; ^∧^*p* < 0.05 vs. female VIC of same phenotype by two-way ANOVA with Tukey's post *hoc* test. *N* = 3 pigs per sex, *n* = 3 wells per condition.

The suppressive effects of VIC-conditioned media on VEC proliferation led us to postulate that the media may be enriched in anti-proliferative factors. Thrombospondin-2 (TSP2) and chrondromodulin-1 (Chm1) are known to be anti-angiogenic molecules (8, 20, 21), so we evaluated their presence in our VIC cultures. TSP2 secretion was highly dependent upon both VIC sex and phenotype. Greater amounts of TSP2 were secreted by female VICs relative to males, and TSP2 production was elevated by approximately 2-fold in aVICs of both sexes, compared to their qVIC counterparts (Figure 5A). The distribution of TSP2 across media and lysate fractions was similar across all four conditions (Figure 5B). Chm1 was detected only in the culture lysate and was found to be decreased in aVIC cultures, without exhibiting any sex-dependence (Figure 5C).

### VIC-Secreted TSP2 Is Partially Responsible for Suppressing VEC Proliferation

**Figure 6:**
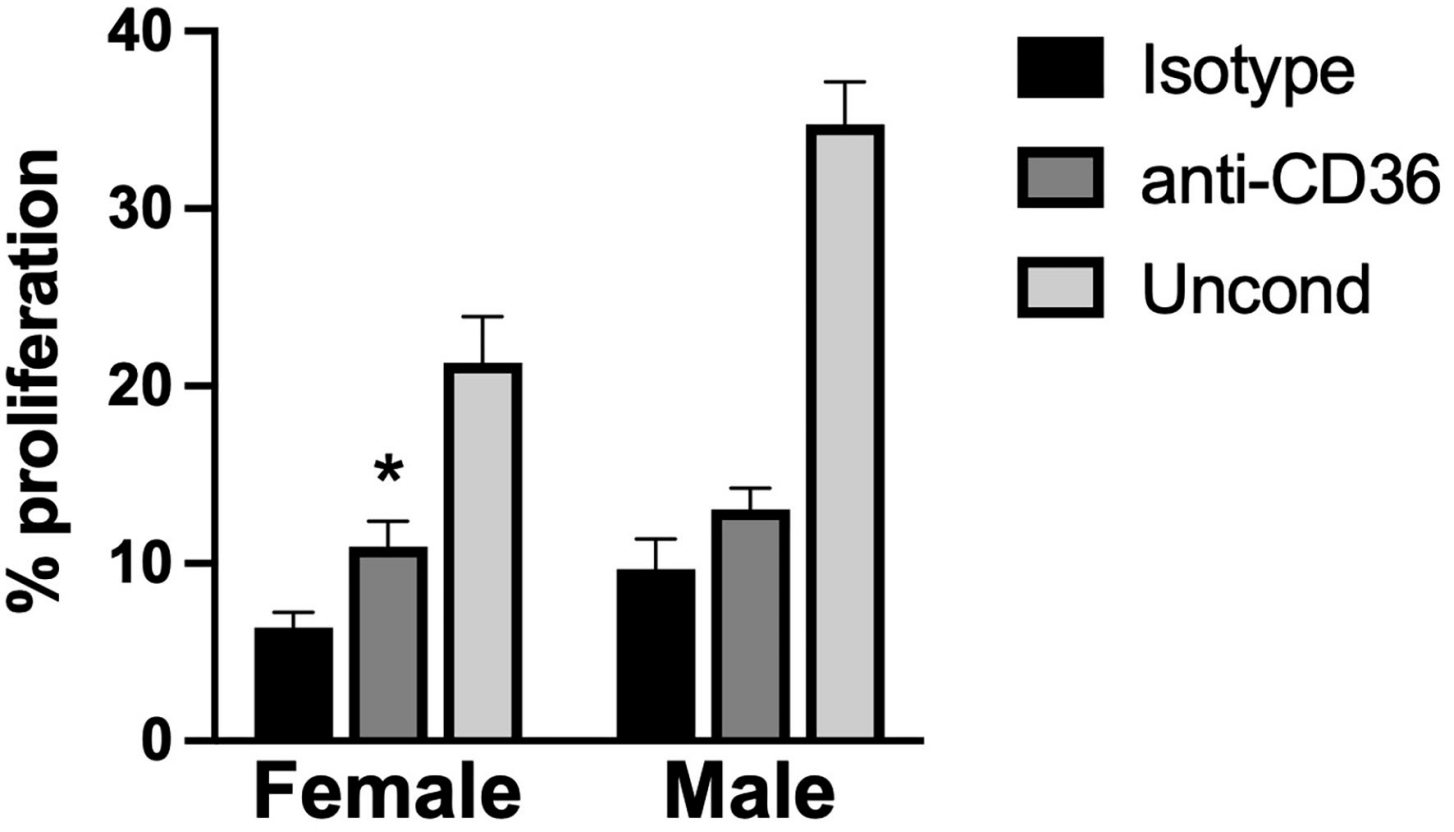
VEC proliferation upon blocking TSP2 in VIC-conditioned media. VEC proliferation was measured following treatment with an antibody against CD36, to block VEC recognition of TSP2, or an isotype control. *p < 0.05 vs. sex-matched isotype control. *N* = 3 pigs per sex, *n* = 3 wells per condition.

Observationally, it appeared that levels of TSP2 in the media (Figure 5A) inversely correlated with the proliferation trends seen with conditioned media (Figure 4B). Thus, we sought to understand whether this factor was responsible for driving the suppression of VEC proliferation seen upon treatment with aVIC-conditioned medium. There are no small molecule inhibitors of TSP2 itself, so a blocking antibody to CD36, the receptor that recognizes TSP2, was used to block cellular recognition of the TSP2 in VIC-conditioned medium. As seen in Figure 6, blocking CD36 partially recovered VEC proliferation compared to the isotype control in the female condition. The blocking was effective only in females, which is also where we had found higher levels of TSP2 in the conditioned medium.

## Discussion

The precise role of angiogenesis in CAVD is not yet understood, but it is hypothesized to promote CAVD pathogenesis by contributing to the recruitment of inflammatory cells (22), and could potentially serve as a target for slowing the progression of fibrocalcific events (11). Our characterization found that VIC expression of a myofibroblastic phenotype was associated with increased expression of many angiogenesis-related factors. Moreover, although sexual dimorphism in CAVD has been recognized (4, 5, 23, 24), few studies have characterized cellular-scale sex differences in valvular cells. In this work, we found intrinsic differences in the growth factor secretion profile of male and female VICs, as well as the proliferative behaviors of VECs. Overall, our results do not indicate a single dominant VIC-secreted factor in regulating angiogenic VEC outcomes, but they do point toward VIC phenotype being highly influential with respect to modulating angiogenesis on both the molecular and functional (e.g., VEC behavior) scale. Cellular-scale sex also significantly influenced VIC angiogenic secretion profiles, but it was ultimately associated with only modest changes in VEC behavior outcomes.

In the early stages of valve thickening, the majority of VICs are thought to still be in a qVIC phenotype (25). However, during later stages of CAVD, when neovascularization becomes evident, activated VICs (aVICs) represent the dominant phenotype. The potential contribution of different VIC phenotypes to angiogenic events is not well understood, as the ability to intentionally generate and maintain qVIC cultures is a relatively recent development (13, 14). In previous work, *ex vivo* cultures of small valve explants revealed that more capillary-like outgrowths occurred in stenotic valve cultures compared to non-stenotic (26). A recent study also showed that VICs from valves with CAVD were more pro-angiogenic than VICs from healthy valves (11). Although that study did not explicitly culture the VICs from healthy valves as qVICs, their finding that diseased VICs secreted more VEGF into the media than healthy VICs was consistent with our results showing increased secretion of VEGF by aVICs compared to qVICs.

However, in contrast with the aforementioned VIC angiogenesis study, our results indicated that media conditioned by pathological VICs attenuated endothelial cell proliferation compared to media from healthy VICs (11). This difference between studies may be affected by using a different endothelial cell source, where the prior work used endothelial colony forming cells (ECFCs) isolated from umbilical cord blood, while our endothelial cells were adult VECs. Differences in donor age and type of cell may contribute to variations in their proliferative and angiogenic capacities and responses to stimuli. Additionally, the valves and VICs in all previous work were pooled from both males and females, so sex differences could not be examined. With respect to the decreased VEC proliferation seen in our work, we hypothesize that it was due to a combination of decreased bFGF and increased anti-proliferative factors (of which TSP2 is only one such molecule) in the aVIC media. It is important to note that the proliferation of VECs induced by aVIC-conditioned media was not just lower than the qVIC condition, but also lower than the negative (non-conditioned) control. This finding suggested that there was an anti-proliferative force at play, rather than just differences in bFGF, which caused us to focus our follow-up efforts on TSP2. Blocking the receptor for TSP2 was found to modestly recover VEC proliferation in the female aVIC-conditioned medium, indicating that the high TSP2 levels in female aVICs were partially responsible for the anti-proliferative effects. Because this study focused on examining factors that were primarily associated with angiogenesis, Chm-1 and TSP2 were the first molecules we investigated in the context of negative VEC regulation. However, there are many anti-proliferative molecules secreted by VICs that are not necessarily prominent in angiogenesis, and our previous work revealed sexual dimorphism in VIC gene expression of some of these (e.g., serpinB2, apoliprotein E) (12). Our current findings motivate further study into VIC secretion of such proliferation-regulating molecules and their sex-dependent impact on VECs.

Many of the trends observed in our study are consistent with histological observations of human valves with CAVD. For example, VEGF and ET-1 are elevated in sclerotic valves (27), which aligns with our finding of increased VEGF and ET-1 production by aVICs compared to qVICs. Studies of human plasma also indicate higher levels of ET-1 in males compared to females (28), which is matched by our VIC observations. TSP2 is upregulated in CAVD (29) while chondromodulin-1 is downregulated (30), which also mirrors our *in vitro* trends. One outlier in our findings was bFGF, in that it was the only pro-angiogenic factor we tested that decreased in aVICs. It is not known how this compares to the case of human valves, as it does not appear that diseased human valves have been tested for bFGF. In our work, although the media used for qVIC and aVIC experiments was not supplemented with bFGF, it is possible that the previous exposure of qVICs to bFGF-supplemented media during qVIC expansion induced a lasting upregulation of endogenous bFGF secretion, since bFGF is known to induce its own expression (31).

The current study also examined the total growth factor content of VIC cultures (i.e., not only growth factor present in the media), which has not previously been characterized. For all growth factors examined, the majority of the biomolecule was present in the lysate fraction, which represents growth factors that had been secreted and were bound to the ECM, as well as growth factors that were still located in the intracellular space. One interesting outcome that emerged was a lesser fraction of growth factor in male VIC lysates compared to females. This difference motivated us to examine whether male and female VICs differed in their production of the ECM component that is most commonly responsible for growth factor sequestration. In fact, male VICs produced less HSPG2 than female VICs, which could contribute to the lower abundance of growth factors in male VIC lysates. Differences in ECM production by male and female aVICs have not previously been documented, but have the potential to affect numerous processes related to CAVD progression.

Although the molecules examined in this study are commonly associated with angiogenesis, regulation of this behavior is not their only role. Notably, several of the molecules that we quantified are also known to be potent regulators of fibrosis. For example, TSP2 is known to be powerfully pro-fibrotic (32), as it regulates molecules related to collagen fibrillogenesis, crosslinking, and degradation (33, 34). Thus, while its upregulation in diseased cells and tissues may seem counterintuitive from an angiogenesis perspective, it does align with the increase in fibrosis seen in CAVD. Our TSP2 findings are particularly interesting in the context of CAVD sexual dimorphism, as TSP2 levels were over 2-fold higher in female aVICs compared to male aVICs, a trend that aligns with fibrosis being more prevalent in female valves compared to male valves (4). In fact, the largest sex differences observed across all molecules quantified in the present study were found in TSP2. Thus, although our data related to angiogenesis did not reveal very strong sex-related associations, our TSP2 findings may have implications for understanding sexual dimorphism in valvular fibrosis.

Of course, this study is not without limitations. The VICs were cultured on 2-dimensional tissue culture polystyrene, which does not mimic the physical or biochemical properties of the native valve. It is possible that the growth factor secretion profiles may differ in ECM-based environments with different biophysical properties (35). Moreover, although porcine valves exhibit a high degree of similarity to human valves, the angiogenic behavior of human valve cells may differ from the porcine valve cells studied herein. Also, while numerous growth factors relevant to angiogenesis were quantified in this work, there may be other influential pro- or anti-angiogenic biomolecules that are produced by VICs. This study also raises several avenues to pursue in future work regarding CAVD angiogenesis and sexual dimorphism. For example, macrophages are known to be a potent driver of angiogenic processes (36), so VICs are unlikely to be the only source of angiogenic factors in the context of the diseased valve. Once the valve has been infiltrated by inflammatory cells, it is possible that VICs may play a lesser role in driving angiogenesis. Another issue raised by this work is the existence of sexual dimorphism in VEC function and how it may affect valvular susceptibility to disease. Cellular-scale sex differences have been documented in ECs from other tissue sources (37); the current work examined only a few VEC functions, but motivates an expanded study on this topic for future work. Finally, the mechanism for the cellular-scale sex differences noted herein is not yet known. Potential hypotheses to examine in future work include chromosome complement or cellular memory of sex hormones (38).

## Data Availability Statement

The original contributions presented in the study are included in the article/supplementary material, further inquiries can be directed to the corresponding author.

## Author Contributions

KS, CM, and KM conceived of the ideas. VN, VP, LS, KS, CM, and KM had various roles in designing and performing the experiments. VN, VP, and KM analyzed the data. All authors approved of the manuscript.

## Conflict of Interest

The authors declare that the research was conducted in the absence of any commercial or financial relationships that could be construed as a potential conflict of interest.

## Publisher's Note

All claims expressed in this article are solely those of the authors and do not necessarily represent those of their affiliated organizations, or those of the publisher, the editors and the reviewers. Any product that may be evaluated in this article, or claim that may be made by its manufacturer, is not guaranteed or endorsed by the publisher.
